# Singing Voice Detection: A Survey

**DOI:** 10.3390/e24010114

**Published:** 2022-01-12

**Authors:** Ramy Monir, Daniel Kostrzewa, Dariusz Mrozek

**Affiliations:** Department of Applied Informatics, Silesian University of Technology, 44-100 Gliwice, Poland; ramy.monir@polsl.pl (R.M.); dariusz.mrozek@polsl.pl (D.M.)

**Keywords:** singing voice detection, vocal detection, music information retrieval, hidden Markov models, support vector machines, Mel-frequency cepstrum coefficients, perceptual linear prediction, short-time Fourier transform, deep learning models, datasets

## Abstract

Singing voice detection or vocal detection is a classification task that determines whether there is a singing voice in a given audio segment. This process is a crucial preprocessing step that can be used to improve the performance of other tasks such as automatic lyrics alignment, singing melody transcription, singing voice separation, vocal melody extraction, and many more. This paper presents a survey on the techniques of singing voice detection with a deep focus on state-of-the-art algorithms such as convolutional LSTM and GRU-RNN. It illustrates a comparison between existing methods for singing voice detection, mainly based on the Jamendo and RWC datasets. Long-term recurrent convolutional networks have reached impressive results on public datasets. The main goal of the present paper is to investigate both classical and state-of-the-art approaches to singing voice detection.

## 1. Introduction

The singing voice is an essential component of music, serving as a communication channel for lyrics and rich emotions. A high level of expressiveness of human singing is even considered ideal for the instrument player to aspire toward. The human vocal apparatus generates sound by moving air forced by the diaphragm through the vocal folds, causing them to vibrate. Modulating airflow through the vibrating vocal folds produces a wealth of different timbres. Timbre is independent of the perceived pitch of a tone. It allows us to distinguish between vowels and consonants in words and the distinct sound qualities of various musical instruments.

Since human voice detection in music tracks is the basis for many advanced applications, it has been studied for several years. In the field of music information retrieval, singing voice detection (SVD) is the preprocessing step that can be used to improve the performance of other tasks such as automatic lyrics alignment [1,2,3], singing melody transcription [4,5], singing voice separation [6,7,8], vocal melody extraction [9], lyric transcription [10,11], singer identification [12], etc.

To the best of our knowledge, no recent review article has been written on the singing voice detection problem. As a result, in this paper, we would like to fill this gap, and we investigate the classical approaches of SVD systems [13] which focus on the acoustic similarity between singing voice and speech, using cepstral coefficients [13] and linear predictive coding [14]. In addition, we review the existing SVD systems with the use of machine learning classifiers such as random forests, artificial neural networks, and support vector machines combined with a large set of audio descriptors (e.g., spectral flatness) as well as special features such as fluctograms [15]. Lastly, we review the state-of-the-art techniques using deep neural networks, which the SVD systems can apply to learn features using a recurrent neural network (RNN) [16] and convolutional neural network (CNN) [17]. Lately, new types of neural network structures have been widely applied to solve many difficult tasks [18]. There is a difference between a human singing voice and regular speech (speaking voice), mainly in intonation manipulation. Yasunori et al. [19] proposed two models to differentiate between a singing voice and a speaking voice based on Mel-frequency cepstrum coefficients (MFCCs). A singing voice utilizes vocal cord muscle tension to regulate the pitch and duration. Its average intensity is thus beyond that of speech, its dynamic vary is more significant, and its tone is usually totally different from that of speech [20].

In order to locate vocal segments, researchers usually extract one or more types of features from the audio signals and then use the classifier to detect them [18]. There are various types of attributes, but MFCCs and the spectrum obtained with short-time Fourier transform (STFT) were the most commonly used features for the SVD task. The features and statistical classification methods used in speech recognition have some limitations in detecting singing voices. Deep learning, with its powerful feature representation as well as time and space modeling capabilities, has recently begun to be used in singing voice detection [21].

To detect the singing voice in music tracks, researchers usually split the speech signal into three portions: voiced (a strong sound in which the vocal cords vibrate), unvoiced, and silent parts. There are several voiced and unvoiced regions in speech. If the system’s input excitation is a nearly periodic impulse sequence, the corresponding speech appears visually nearly periodic and is referred to as voiced speech. While the excitation is random noise-like, the resulting speech signal is random noise-like as well, with no periodic nature, and is referred to as unvoiced speech. The classification of speech signals as voiced or unvoiced provides a preliminary acoustic segmentation for speech processing applications such as speech synthesis, speech enhancement, and speech recognition [22].

This paper is organized as follows: Section 2 is focused on feature extraction, and Section 3 presents the most used datasets for SVD. Section 4 gives the outline of the existing, classical methods for SVD. Section 5 describes the state-of-the-art methods for SVD, and the paper is concluded in Section 6.

## 2. Feature Extraction

Singing voice detection is a crucial task that can be used to improve other tasks such as automatic lyrics alignment, singing melody transcription, vocal melody extraction, lyric transcription, singer identification, etc. To analyze music presence in a recorded audio signal, a representation that roughly corresponds to how people perceive sound through their auditory system has to be created. At a fundamental level, such audio representations aid in determining when events occur in time [23].

In order to locate vocal segments, researchers usually extract one or more types of features from the audio signals and then use a classifier to detect these audio features. The feature extraction stage is therefore critical for the subsequent classification process. Using a feature set (combining multiple features) usually results in better performance. Audio features provide the description of the sound that helps capture different aspects of sounds and build intelligent audio systems.

Audio features can be applied to feature extraction linked to audio effects [24], data classification [25], similarity measures [26], data mining [23], and feature-based synthesis [27], etc. Audio features can be categorized into three levels of abstraction: low-level such as spectral centroid, spectral flux, energy, zero-crossing rate; mid-level such as MFCCs; and high-level audio features such as lyrics, melody, and rhythm [28].

*Short-Time Fourier Transform Spectrum*

The Fourier transform is a mathematical formula for decomposing a signal into its individual frequencies and amplitudes. To put it another way, it converts the signal from the time domain to the frequency domain to create a spectrum. Perhaps short-time Fourier transform (STFT) spectrum is the most common time-frequency representation and has been widely used in various domains other than music processing. The STFT is also used to represent other audio features such as Mel-frequency cepstral coefficients (MFCCs) and chroma features [23].

*Mel-spectrogram*

The Mel-scale is a perceptual scale of pitches. A spectrogram is a visual image of a signal’s frequency spectrum as it changes over time. A spectrogram is obtained by applying STFT on overlapping windowed segments of the signal. This spectrogram is a graphical way of representing STFT data. Mel-spectrogram is often used when applying deep learning approaches because it is more efficient than STFT spectrum [29].

*Temporal Features*

Temporal features describe a music signal’s relatively long-term dynamics over time [30]. They are basically time-domain features, such as amplitude envelope, the energy of the signal, root mean square energy, zero-crossing rate (ZCR), etc., which are easy to extract. ZCR counts how many times the signal changes sign from negative to positive or vice versa in a specified time frame (in seconds). In the process of speech recognition and music information retrieval, ZCR is an essential feature in voice/noise classification. Speech can be unvoiced, and voiced fricatives (speech) have higher ZCR.

*Spectral features*

Spectral features, such as band energy ratio, spectral centroid, bandwidth, spectral roll-off, Mel-frequency cepstral coefficients (MFCC), perceptive linear prediction (PLP), linear prediction cepstral coefficients (LPCCs) [31], etc., are frequency domain features that are derived by converting the time domain into the frequency domain using the Fourier transform. The spectral features can be used to determine the rhythm, notes, pitch, and melody. Spectral centroid calculated as the weighted average of the frequencies in the signal is determined by a Fourier transformation with their magnitudes as weights. The spectral centroid is used to calculate a sound’s brightness, and it is an important factor in describing musical timbre.

**Figure 1:**
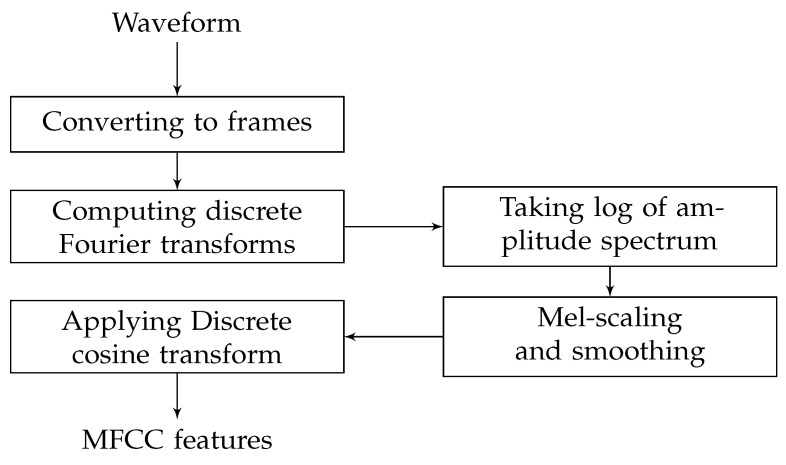
Calculation steps for MFCC features [35].

MFCCs are widely used in SVD [32] and were first introduced by Davis and Mermelstein in 1980 [33]. Kim et al. [34] compared MFCC and audio spectrum projection features, and they mentioned that MFCCs were better for feature extraction. The use of MFCCs has proven to be a powerful tool in music and voice recognition, and sound recognition in general. The MFCCs are calculated as follows:

(1) $$C\left(x\left(T\right)\right)=F^{-1}\left[log\left(F\left[x\left(t\right)\right]\right)\right]$$

where x(t) is the time-domain signal. Figure 1 shows the steps to compute MFCC features.

Calculation of the MFCC includes the following steps:

1. Division of the speech signals into frames, usually by applying a windowing function at fixed intervals [35];
2. Computing the coefficients of the discrete Fourier transform on each segment of windowed signal to convert the time domain into the frequency domain;
3. Taking the logarithm of the amplitude spectrum;
4. Smoothing the spectrum and emphasizing perceptually meaningful frequencies [35];
5. Taking the discrete cosine transform (DCT) of the list of mel log powers;
6. Generating cepstrum.

## 3. Datasets

This section outlines the most commonly used datasets to perform the SVD task. Most scientific papers related to the SVD task use small designed datasets such as Jamendo Corpus, RWC Popular Music, MedleyDB, MIR-1k, and iKala. This is due to the fact that it is better to have good quality reference datasets in order to achieve high accuracy in SVD tasks [36].

Table 1 describes the most commonly used datasets in the singing voice detection task and the related papers used for each dataset. Jamendo Corpus and RWC Popular Music datasets are the most popular for SVD.

**Jamendo Corpus** is a public dataset consisting of 93 copyright-free songs. It was introduced by Mathieu Ramona et al. in [48]. It has vocal activation annotations. Moreover, for each song, the segments are annotated as “voice” or “no voice”. The audio files in Jamendo are stereo. The dataset is divided into a training set containing 61 files, a validation set of 16 files and a test set of 16 files.

**RWC Popular Music** is a public dataset consisting of 100 pop songs with vocal activation and instrument annotations. It was introduced by Mauch et al. in [49]. It contains 80 Japanese songs and 20 English songs. The audio files in RWC are stereo and have a sampling frequency of 44.1 kHz and 16 bits per sample.

**MedleyDB** is a multitrack dataset containing 122 tracks. It was introduced by Bittner et al. in [50]. It does not provide annotations for vocal or nonvocal segments, but it includes instrument activations, genre, and melody annotations. The audio format is WAV and has a sampling frequency of 44.1 kHz with 16 bits per sample.

**MIR-1k** (Multimedia Information Retrieval lab) consists of 1000 songs with vocal activation and pitch contours annotations. MIR-1k was introduced by Chao-Ling Hsu et al. in [51]. For each song, the segments are annotated as “voice” and “no voice”. The sampling rate is 16 kHz, and for all the 1000 clips, the clip duration is 4–13 s.

**iKala** dataset contains 352 30-seconds clips of Chinese popular songs. It was introduced by Chan et al. [52]. This dataset includes nonvocal regions and has a sampling frequency of 44.1 kHz.

## 4. Traditional Methods

The singing voice detection task was first proposed by Berenzweig and Ellis in [13]. The authors focused on the problem of recognizing singing segments in popular music as a valuable and tractable method of music content analysis, and they used the statistical features and the hidden Markov model as a classifier. They were able to derive various statistics and models using Posterior Probability Features obtained from the acoustic classifier of a general-purpose speech recognizer. This approach enabled them to train an effective SVD system that was around 80% accurate at the frame level.

Namunu et al. [53] presented an approach for detecting singing voice boundaries derived from acoustical polyphonic music signals. They called this approach twice-iterated composite Fourier transform (TICFT). First, the music signal was divided into frames based on quarter notes. The harmonic structure of each frame was then measured using TICFT. Finally, the vocal and instrumental frames were classified using music domain knowledge. They mentioned that this method is less complex and more accurate than statistical learning methods. In terms of vocal boundary detection, they achieved over 80% frame-level accuracy.

Vembu et al. [54] presented a technique to identify vocal parts in music samples. They designed a classifier to perform a vocal–nonvocal segmentation task. They trained a neural network using several features: MFFCs, perceptual linear prediction (PLP), and log frequency power coefficients (LFPC), achieving the accuracy of 84.87% for the singing voice segmentation task. In [55], Lukashevich et al. used the autoregressive moving average model as a postprocessor reaching the accuracy of 82.5%.

In [32], Rocamora and Herrera used various existing statistical descriptors and studied the accuracy of estimating vocal segments in music audio. They compare MFCCs, PLPs, LFPCs, and the harmonic coefficient (HC). The most appropriate feature was MFCC, and the best-performing classifier was the support vector machine. They also considered spectral features commonly used for instrument classification, such as centroid, roll-off, flux, skewness, kurtosis, and flatness. They reached the classification accuracy of 78.5% on the Jamendo dataset. In [15], Dittmar et al. suggested combining MFCCs with fluctogram variation and vocal variation. The authors used the random forest as a classifier and obtained the F-measure at the level of 87%.

In [7], Li and Wang used a singing voice detection step before separating the vocals from the instrumental accompaniment. The authors used several features (MFCCs, linear prediction coefficients—LPC, PLP, and the 4-Hz harmonic coefficient) and fed to a hidden Markov model (HMM) combined with the Viterbi algorithm [56]. Their 10-fold crossvalidation setup was based on only five rock and five country songs semiautomatically annotated from a karaoke CD. For training, several versions of each song with varying levels of signal-to-noise ratio (SNR) were generated, which was a type of data augmentation that has grown in popularity in recent years. The authors reported the accuracy of 80%, 85%, 90%, and 92%, respectively, for –5, 0, 5, and 10 dB SNR.

Hsu et al. [51] proposed a singing voice separation system to identify and separate the unvoiced parts from the music accompaniment. The first stage of the system was singing voice detection. To decode music signals into the three groups—accompaniment, unvoiced, and voiced—the authors used hidden Markov models (HMMs). Then, they used Gaussian mixture models (GMMs) as states in a fully connected HMM and the Viterbi algorithm. In another work, Hsu et al. [43] used SVD for pitch estimation and vocal separation. They trained two GMMs to model vocal and nonvocal classes. The Viterbi algorithm was then used to decode the GMMs as states in a fully connected, continuous HMM.

In [48], Ramona et al. used several features such as MFCCs, ZCR, and sharpness. After a silent detection stage, these features were fed into a support vector machine as a classifier. On the output of the predicted sequence, the authors proposed a temporal smoothing strategy considering the temporal structure of the annotated segments. Instrumental portions less than 0.5 seconds in length were also smoothed out. On the publicly accessible Jamendo dataset, they reported accuracy of 82.2%, as well as precise information on training and test set split.

In [38], Regnier et al. extracted sinusoidal partitions from musical audio signals and analyzed frequency modulation (vibrato) and amplitude modulation (tremolo) of each partition. They reached the accuracy of 76.8% by applying thresholds for vibrato and tremolo. A more advanced approach involving numerous characteristics and a GMM as a classifier result in a 77.4% for the F-measure.

Lehner et al. [39] proposed a real-time-capable and straightforward method to detect the presence of a human voice in audio signals. They used only MFCCs for feature representation and random forest as a classifier. They achieved an accuracy of 82.3% after the final optimization of the classifier parameters.

## 5. Deep Learning Techniques

In this section, we discuss the techniques used by researchers in the singing voice detection task with the help of deep learning techniques. Neural networks are widely used for solving this problem, and one of the recurrent neural network types, namely, the long short-term memory network, has been widely used by many researchers in SVD.

### 5.1. Convolutional Neural Networks

Convolutional neural networks (CNNs) are similar to traditional artificial neural networks (ANNs) in that they are made up of neurons that optimize themselves through learning. Each neuron continues to receive input and perform an operation (such as a scalar product followed by a nonlinear function), which is the foundation of many ANNs [57].

Schlüter et al. [17] introduced a model for singing voice detection using CNN. They used three-by-three 2D convolution layers. The model is capable of learning invariance by data augmentation. In the training phase, the authors applied data augmentation, such as time stretching and pitch shifting, on the audio representation. They developed several augmentation methods that can be efficiently used to work on spectrograms or Mel-spectrograms. Two of the augmentation techniques are data independent, while four are audio data specific, and one is specific to binary sequence labeling.

You et al. [18] applied the CNN model for singing voice detection with MFCC features, fast Fourier transform (FFT) features, raw pulse-code modulation (PCM) samples, and long short-term memory. They called it CNN for MFCC feature (MCNN), CNN for spectrogram (SCNN), end-to-end CNN for raw PCM samples (ECNN), and convolutional LSTM (CLSTM). MCNN, SCNN, CLSTM, and ECNN were trained and tested using the Jamendo Corpus dataset and achieved the accuracy of 88.2%, 91.8%, 77.1%, and 90.4%, respectively. SCNN achieved the best accuracy after ten trials on the Jamendo dataset.

Huang et al. [58] proposed various structures of CNN for SVD. The input features were MFCC, discrete Fourier transform (DFT) coefficients, and raw PCM samples. The authors found out that DFT coefficients achieved higher detection accuracy (up to 92%) evaluated on all epochs over the average of 10 trials which is higher than MFCC and raw PCM.

In [59], Wenming Gui et al. have significantly improved CNN presented in [29] by adding batch normalization, changing the activation function to Leaky ReLU, and analyzing attention distribution of the feature maps. The numerical results were achieved on Jamendo Corpus, RWC Popular Music, and MIR-1k datasets.

Krause et al. in [60] analyzed the generalization capabilities and robustness of two models in a different scenario. They used opera recordings as a dataset. The studies were performed for one standard classifier—random forest—and one based on deep learning technique—CNN [15]. The quantitative results have shown that CNN outcomes are slightly better than those obtained with the random forest classifier.

### 5.2. Recurrent Neural Networks

A recurrent neural network (RNN) is a computational neural network with feedback connections. RNNs can deal with time-series signals such as audio and video effectively and flexibly [61]. In a simple RNN, the hidden state at a time *t* is computed as follows:

(2) $$h_{t}=f\left(W_{ih}i_{t}+W_{hh}h_{t-1}+b_{h}\right)$$

(3) $$z_{t}=f\left(W_{hz}h_{t}+b_{z}\right)$$

where *f* is an activation function; $h_{t}\in R$ is the hidden state with *N* hidden units; $W_{ih}$ represents weight matrices of connections between input and hidden layers; $i_{t}$ is the input at time *t*; *b* denotes the bias vector; $z_{t}$ is an output vector; and $W_{hh}$ represents weight matrices of connections between hidden and hidden layers.

RNNs are used to process sequential data in such a way that each data point can be understood in a context. RNNs have demonstrated success in tasks such as text generation [62] and speech recognition [63]. They can be used to model nonlinear sequential relationships, but it is hard to train a simple RNN due to vanishing gradient and exploding problems, and the problem of long-term dependencies.

Hughes et al. [64] proposed a recurrent neural network model for voice activity detection. The model is multilayered, where the nodes compute quadratic polynomials, and all proposed model parameters are optimized together. The authors have shown that the proposed model can outperform larger GMM-based systems on voice activity detection tasks.

### 5.3. Long Short-Term Memory

Long Short-Term Memory (LSTM) is a special kind of RNN that can learn long-term dependencies. Moreover, it is designed to avoid the long-term dependency problem. Remembering information for extended periods is practically LSTM’s default behavior [65].

**Figure 2:**
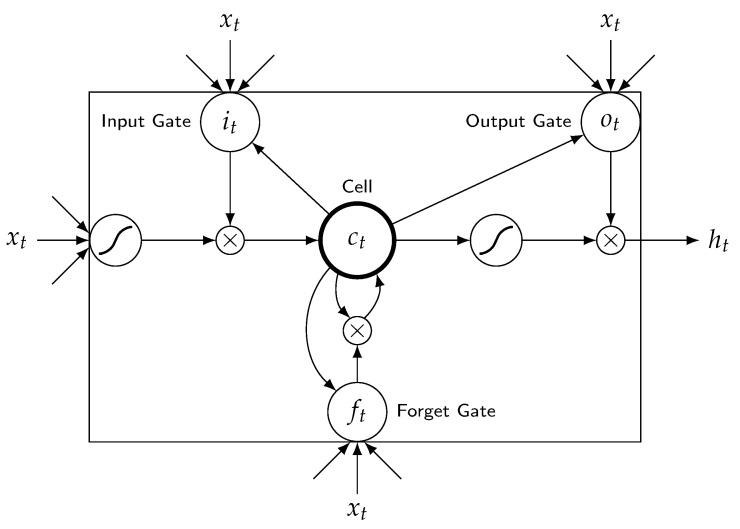
LSTM cell.

As presented in Figure 2, each LSTM block includes a memory cell. The input and forget gates monitor the content of the network while it is performing classification at each time level. The input of the block to which it belongs can be stored in the cell for as long as it is required. LSTM cell can be described as follows:

- $\begin{array}{c} \mathrm{Forget}\mathrm{gate}:\;f_{t}=\sigma \left(W_{f}.\left[h_{t-1},x_{t}\right]+b_{f}\right) \end{array}$
- $\begin{array}{c} \mathrm{Input}\mathrm{gate}:\;i_{t}=\sigma \left(W_{i}.\left[h_{t-1},x_{t}\right]+b_{i}\right)\\ {\tilde{C}}_{t}=tanh\left(W_{c}.\left[h_{t-1},x_{t}\right]+b_{C}\right) \end{array}$
- $\begin{array}{c} \mathrm{Cell}\mathrm{state}:\;C_{t}=f_{t}*C_{t_{1}}+i_{t}*{\tilde{C}}_{t} \end{array}$
- $\begin{array}{c} \mathrm{Output}\mathrm{gate}:\;i_{t}=\sigma \left(W_{o}.\left[h_{t-1},x_{t}\right]+b_{o}\right)\\ h_{t}=o_{t}*tanh\left(C_{t}\right) \end{array}$

where “.” is the element-wise product, $i_{t}$ represents the input gate, $f_{t}$ represents the forget gate, $o_{t}$ represents the output gate, σ represents the sigmoid function, $w_{x}$ is the weight for the respective gate (*x*), $h_{t-1}$ is the output for the previous LSTM block at (t−1) timestamp, $x_{t}$ is an input at current timestamp, and $b_{x}$ are biases for the respective gates (*x*).

A typical LSTM cell has three gates: a forget gate, an input gate, and an output gate. The forget gate of the LSTM cell determines how much of the previous data should be forgotten. The input gate determines how much information is written to the internal cell state. The output gate determines the next hidden state to be generated from the current internal cell state. LSTM units have a single memory cell that allows them to store data for an indefinite period. This memory cell’s read, write, and delete operations are handled by gates that function similarly to standard units.

Not only are the hidden units connected to the input units (or, in the case of consecutive hidden layers, to the units of the preceding hidden layer), but each unit is also connected to itself, i.e., the previous time step. The RNN has access to past information via these recurrent connections to model temporal context.

Using LSTM-RNN seems to be one of the best choices for singing voice detection. Eyben et al. [66] presented a data-driven method to voice activity detection trained on RASTA-PLP as front-end features based on long short-term memory-recurrent neural network (LSTM-RNN). The results show that LSTM-RNN outperforms all other methods in statistical benchmarks.

Lehner et al. [37] used LSTM-RNN for singing voice detection. They applied a unidirectional RNN with one hidden layer and 55 LSTMs to sort out frames into vocal and nonvocal. The input was based on several audio features in the feature representation that included 30 MFCCs. The authors achieved state-of-the-art performance on Jamendo and RWC datasets.

### 5.4. Bidirectional LSTMs

Bidirectional RNN (BRNN) are simply two separate RNNs joined together. The idea behind BRNN is to divide the state neurons of a regular RNN into two parts: one for the positive time direction (forward states) and one for the negative time direction (backward states). RNNs can only use a past temporal context. When the entire sequence of input features is available, it can also be possible to take advantage of the future context. This can be accomplished with a bidirectional RNN (BRNN). When it comes to learning long-term dependencies, LSTM-RNNs have proved to be superior to regular RNNs [67].

**Figure 3:**
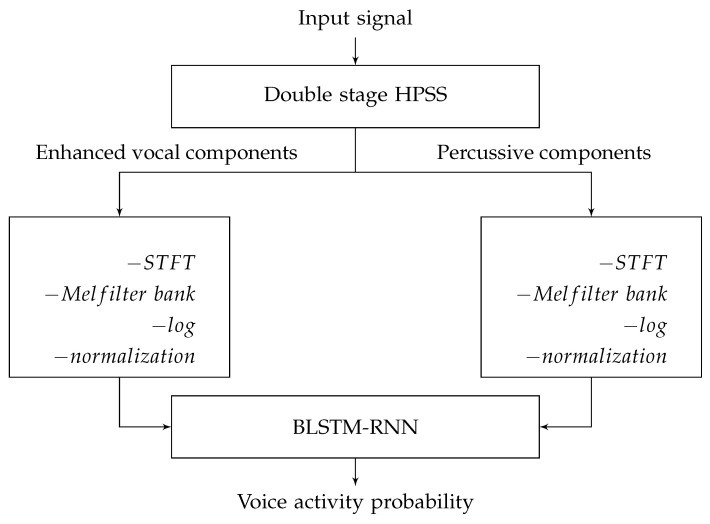
The overview of the BLSTM architecture used for SVD in [16].

Leglaive et al. [16] combined deep BRNNs and LSTM to form deep BLSTM-RNNs and make use of a long-range past and future temporal context in order to classify each input vector. Figure 3 illustrates the system used for this experiment. A system has two-stage harmonic-percussion source separation (HPSS) [68] for the classifier input to extract signals specific to the singing voice. Mel-spectrograms of the obtained harmonic and percussive components are combined as an input for the classifier for each frame. The output predictions for each input frame are produced by several recurrent layers followed by a shared densely connected layer. This classifier can use the inherent sequential aspect of short-term feature extraction in a piece of music to decide on the presence/absence of a singing voice in the past and future temporal context. The authors compared BLSTM with a support vector machine for singing voice segmentation and achieved the accuracy of 91.5% for BLSTM on the Jamendo dataset outperforming other approaches.

### 5.5. GRU-RNN

Cho et al. suggested a gated recurrent unit (GRU) [69] to allow each recurrent unit to capture dependencies across time scales adaptively. The GRU, like the LSTM, has gating units that modulate the flow of information within the unit but without the need for separate memory cells. There are a few differences between GRU and LSTM. GRU reveals its entire content without any monitoring, while LSTM manages the memory content’s exposure—in other words, GRU has a more straightforward structure than LSTM. Another distinction is the addition of new memory content to the system. The update gate is used to monitor information flow in GRU, while the forget gate is used independently in LSTM.

**Figure 4:**
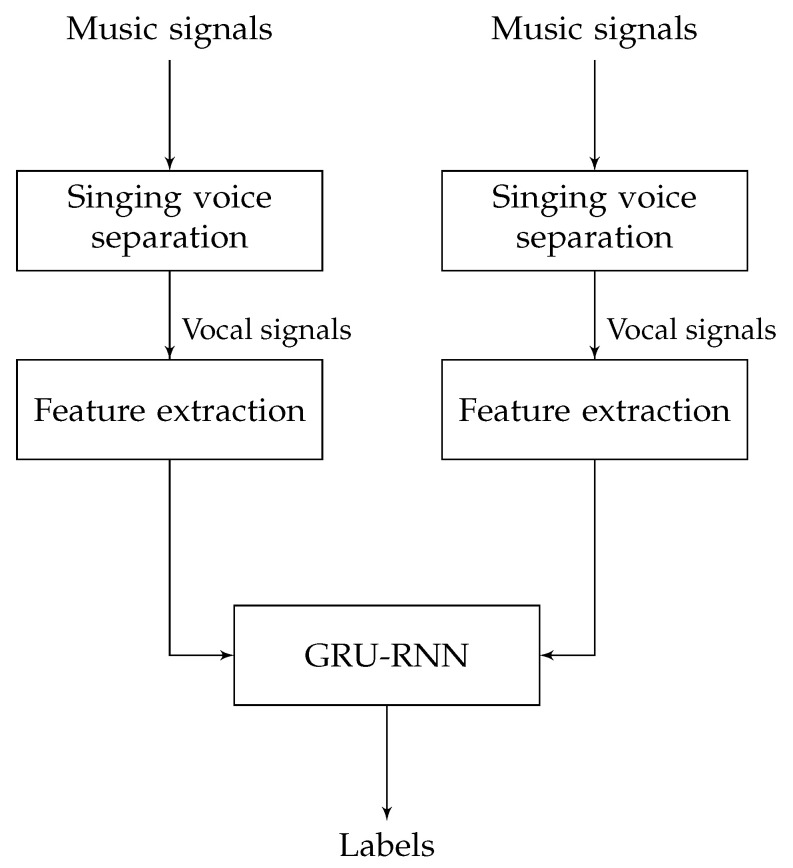
The overview of the GRU-RNN architecture used in [41].

Chen et al. [41] proposed a system (Figure 4) based on GRU-RNN. The preprocessing step used Deep U-Net convolutional networks for singing voice separation. Then, the authors extracted features and fed them to the classifier. The extracted features were MFCC, Mel-filter bank, LPCC, and chroma features. They showed a unidirectional RNN with a hidden layer of 60 GRU units. The classifier’s output is either 1 or 0, with 1 indicating singing and 0 indicating nonsinging. The authors set the block duration as 120 and 720 ms with the temporal smoothing postprocessing step. The authors applied this system for Jamendo and RWC Popular Music datasets. The results are shown in Table 2 and Table 3 for the GRU-RNN (2) with a block duration of 120 ms and GRU-RNN (3) with a block duration of 720 ms.

### 5.6. ConvLSTM or LRCN

Convolutional LSTM (ConvLSTM) networks or long short-term recurrent convolutional networks (LRCNs) have a wide range of applications, including video classification, image captioning, image classification, activity recognition, image labeling, video captioning, singing voice detection, etc. LRCNs can capture both features of CNNs and RNNs by combining them from spatial and temporal features. The LRCN was first proposed in [70]. ConvLSTM is a type of RNN for a spatiotemporal prediction that employs convolutional structures in both input-to-state and state-to-state transitions.

**Figure 5:**
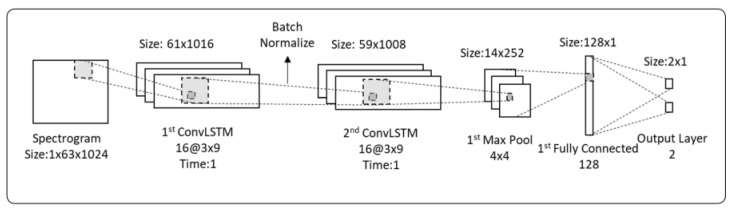
Convolutional LSTM used in [18] (used under the terms and conditions of the Creative Commons Attribution (CC BY) license (http://creativecommons.org/licenses/by/4.0/) (accessed on 10 November 2021)).

You et al. [18] proposed a convolutional LSTM for singing voice detection. The authors mentioned that CLSTM (convolutional LSTM) might theoretically outperform the typical LSTM network with spectrogram inputs because it uses numerous two-dimensional planes as inputs. Figure 5 illustrates the CLSTM network that uses three subplanes as the input sequence.

**Figure 6:**
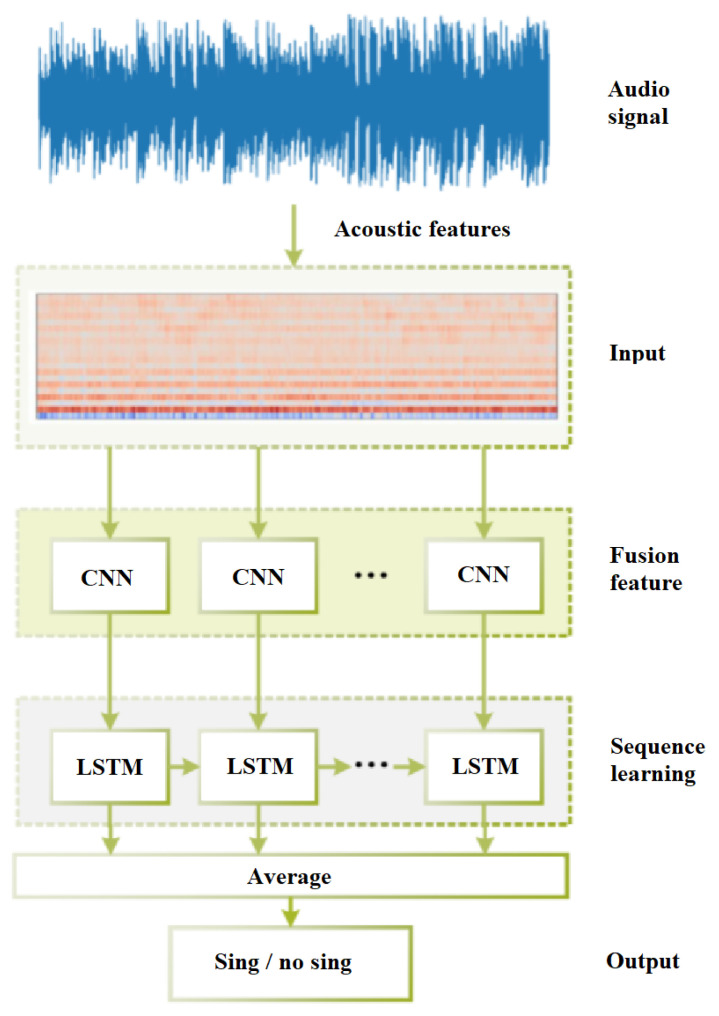
The topology of L RCN used in [21] (used under the terms and conditions of the Creative Commons Attribution (CC BY) license (http://creativecommons.org/licenses/by/4.0/) (accessed on 10 November 2021)).

Zhang et al. [21] proposed LRCN to extract the crucial features that represent the audio content in the frequency domain and characterize the vocal background in the time domain for vocal detection. As presented in Figure 6, the network for detecting singing voices has an input layer that is the same size as the combined acoustic feature vector, three hidden layers, and an output layer with a single sigmoid unit. The network has been trained as a classifier to output vocal scores in a value space of 0 and 1 for each frame-block, where 1 represents a singing voice and 0 represents no singing part of the song. The LSTM layer in LRCN learns the temporal relationship from the features encoded by the convolutional layer. By contrast, the convolutional layer spatially adopts the combined audio features for deep feature extraction. In [21], the proposed system’s architecture employs singing voice separation as a preprocessing technique for obtaining vocal signals. It is then accompanied by a standard classification method, in which the authors applied machine learning techniques (the LRCN) to successive frames of input vocal signals with a collection of audio features. The authors proposed the LRCN model on five different datasets (RWC, Jamendo, MIR-1K, iKala, and MedleyDB), which were mentioned before in Section 3. On the Jamendo dataset, they reached the accuracy of 92% and 0.93 for the f1-score; on the RWC dataset—the accuracy of 97% and 0.96 for the f1-score; on MIR-1K dataset—the accuracy of 94% and 0.89 for the f1-score; on the iKala dataset—the accuracy of 99% and 0.99 for the f1-score; and on the MedleyDB—the accuracy of 81% and 0.79 for the f1-score. They also compared the proposed LRCN model with the existing methods for singing voice detection on the Jamendo Corpus dataset and RWC Popular Music datasets. The results show that LRCN exhibited a state-of-the-art performance.

**Figure 7:**
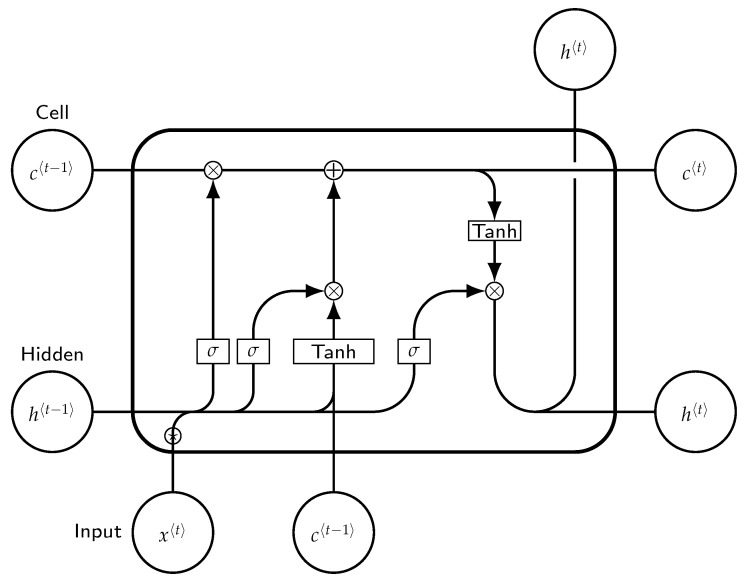
Inner structure of LRCN layer used in [21] (used under the terms and conditions of the Creative Commons Attribution (CC BY) license (http://creativecommons.org/licenses/by/4.0/) (accessed on 10 November 2021)).

Figure 7 illustrates the structure of the LRCN layer. The key equations of LRCN are as follows:

- **Input gate:**$i\left(t\right)=\sigma (W_{i}\cdot \left[Conv\left(X\left(t\right)\right),H\left(t-1\right),C\left(t-1\right)\right]+b_{i})$
- **forget gate:**$f\left(t\right)=\sigma (W_{f}\cdot \left[Conv\left(X\left(t\right)\right),H\left(t-1\right)+C\left(t-1\right)\right]+b_{f})$
- **LRCN Cell:**$C\left(t\right)=f\left(t\right)*C\left(t-1\right)+i\left(t\right)\cdot tanh(W_{c}\cdot \left[Conv\left(X\left(t\right)\right),H\left(t-1\right)\right]+b_{c})$
- **Output gate:**$o_{t}=\sigma \left(W_{o}\cdot \left[Conv\left(X\left(t\right)\right),H\left(t-1\right),C\left(t\right)\right]+b_{0}\right)$
- **Hidden state:**H(t)=o(t)·tanh(C(t))

where “.” is the element-wise product, conv is the convolution operator, $i_{t}$ represents input gate, σ represents sigmoid function, $w_{x}$ is the weight matrix for the respective gate (*x*), $h_{t-1}$ is the output for the previous LSTM block at (t−1) timestamp, $x_{t}$ is an input at current timestamp, and $b_{x}$ are biases for the respective gates (*x*).

Table 2 and Table 3 summarize the existing SVD methods applied on the Jamendo Corpus and RWC Popular Music, respectively. Accuracy is the proportion of correctly classified frames. The recall is the estimated proportion of frames labeled as voiced in the ground truth. Precision is the percentage of frames that are effectively voiced in the ground truth that is measured as voiced by the algorithm. F-measure (also called F1-score) combines the precision and recall of the model and is used to measure the accuracy of the model on a dataset. The number of false-negative (FN), true-negative (TN), false-positive (FP), and true-positive (TP) results accumulated across all songs in the testing set was calculated by comparing model predictions to ground-truth labels. The four evaluation metrics can be represented as follows:

(4) $$Recall=\frac{TP}{TP+FN},$$

(5) $$Precision=\frac{TP}{TP+FP},$$

(6) $$Accuracy=\frac{TP+TN}{totalframes},$$

(7) $$F_{1}=2*\frac{precision*recall}{precision+recall}.$$

The comparison of the SVD methods in Table 2 and Table 3 shows that LRCN and GRU achieved the best results in terms of accuracy, precision, recall, and F-measure.

## 6. Conclusions and Discussion

This paper presents a survey on existing singing voice detection methods. Many possible features can be used for SVD. The long-term recurrent convolutional network achieved state-of-the-art results on both Jamendo Corpus and RWC Popular Music datasets. GRU has a more straightforward structure and higher computational efficiency than LSTM; therefore, GRU achieved better accuracy in the singing voice detection task. We can notice that, by using any algorithm on the Jamendo and RWC, the accuracy on the RWC dataset is always higher than on the Jamendo.

RNNs are good at processing sequence data and making predictions, but they have short-term memory problems. LSTMs and GRUs were developed as a way to reduce short-term memory by using gate mechanisms. Gates are basically neural networks that govern data flow through the sequence chain.

We believe that future works in this field will be focused on the development of bidirectional LSTM, ConvLSTM, and GRU-RNN on DALI [36], Jamendo, and RWC datasets. Researchers will likely turn to knowledge distillation and attention-based mechanisms due to their growing popularity [59,72].

To the best of our knowledge, no recent review article has been written on the singing voice detection problem. As a result, we hope that this research paper can be significant for future research in that it gives a deep understanding of the models and techniques used in SVD.

## Figures and Tables

**Table 1:** Public datasets relevant to singing voice detection.

Name	Number of Tracks	Size	Related Papers
Jamendo Corpus	93	443 mins	[16,17,21,37,38,39,40,41]
MedleyDB	122	437 mins	[21,42]
MIR-1k	1000	113 mins	[21,43]
RWC Popular Music	100	407 mins	[17,21,37,40,41,42,44,45]
iKala	352	176 mins	[21,42,46,47]

**Table 2:** Comparison of existing singing voice detection methods on the Jamendo Corpus dataset.

		Year	Evaluation Measures (in [%])			
Method	Author	Published	Accuracy	Precision	Recall	F-Measure
**SVM**	Ramona [48]	2008	82.2	-	-	84.3
**GMM**	Regnier et al. [38]	2009	-	-	-	77
**Random forest**	Lehner et al. [39]	2013	84.8	-	-	84.6
**Feature Engineering**	Lehner et al. [71]	2014	88.2	88	86.2	87.1
**LSTM-RNN (1)**	Lehner et al. [37]	2015	91.5	89.8	90.6	90.2
**LSTM-RNN (2)**	Zhang et al. [21]	2020	89.5	89.5	89.6	88.8
**CNN (1)**	Schlüter et al. [17]	2015	92.3	-	90.3	-
**CNN (2)**	Zhang et al. [21]	2020	90.4	90.6	90.4	90.3
**CNN (3)**	Gui et al. [59]	2021	88.9	91.4	89.9	90.6
**Bi-LSTMs**	Leglaive et al. [16]	2015	91.5	89.5	92.6	91
**Bootstrapping procedure**	Dittmar et al. [15]	2015	88.2	-	-	87
**GRU-RNN (1)**	Zhang et al. [21]	2020	91	90.8	91.2	91.4
**GRU-RNN (2)**	Chen et al. [41]	2019	88.2	85.39	92.78	88.93
**GRU-RNN (3)**	Chen et al. [41]	2019	90.8	98.2	93.3	91.2
**LRCN**	Zhang et al. [21]	2020	91.6	92.6	93.4	93

**Table 3:** Comparison of existing singing voice detection methods on the RWC Popular Music dataset.

		Year	Evaluation Measures(in [%])			
Method	Author	Published	Accuracy	Precision	Recall	F-Measure
**SVM-HMM**	Mauch [45]	2011	87.2	88.7	92.1	90.4
**Random forest**	Lehner et al. [39]	2013	86.8	87.9	90.6	89.2
**Feature Engineering**	Lehner et al. [71]	2014	87.5	87.5	92.6	90
**LSTM-RNN (1)**	Lehner et al. [37]	2015	92.3	93.8	93.4	93.6
**LSTM-RNN (2)**	X. Zhang et al. [21]	2020	93.7	94.1	93.3	92.8
**CNN (1)**	Schlüter et al. [16]	2015	92.7	-	93.5	-
**CNN (2)**	X. Zhang et al. [21]	2020	94	93.6	94	94.2
**CNN (3)**	Gui et al. [59]	2021	88.9	90.7	97.0	93.7
**GRU-RNN (1)**	X. Zhang et al. [21]	2020	95.2	95.1	95.3	95.3
**GRU-RNN (2)**	Chen et al. [41]	2019	92.1	92.7	95.4	94
**GRU-RNN (3)**	Chen et al. [41]	2019	95.3	96.1	96.9	96.5
**LRCN**	X. Zhang et al. [21]	2020	97	97.1	96.8	96.3

## Data Availability

The data is contained within the article.
